# Effects of mesenchymal stem cells transplantation on cognitive deficits in animal models of Alzheimer’s disease: A systematic review and meta‐analysis

**DOI:** 10.1002/brb3.982

**Published:** 2018-06-06

**Authors:** Meiling Ge, Yunxia Zhang, Qiukui Hao, Yunli Zhao, Birong Dong

**Affiliations:** ^1^ The Center of Gerontology and Geriatrics West China Hospital Sichuan University Chengdu China; ^2^ Collaborative Innovation Center of Sichuan for Elderly Care and Health Sichuan China; ^3^ Chengdu Medical College Chengdu Sichuan China

**Keywords:** Alzheimer’s disease, animal models, cognitive deficits, mesenchymal stem cells, meta‐analysis, morris water maze

## Abstract

**Background:**

Alzheimer’s disease (AD) is a globally prevalent neurodegenerative disease, clinically characterized by progressive memory loss and gradual impairment of cognitive functions. Mesenchymal stem cells (MSCs) transplantation has been considered a possible therapeutic method for Alzheimer’s disease (AD). However, no quantitative data synthesis of MSC therapy for AD exists. We conducted a systematic review and meta‐analysis to study the effects of MSCs on cognitive deficits in animal models of AD.

**Methods:**

We identified eligible studies published from January 1980 to January 2017 by searching four electronic databases (PubMed, MEDLINE, EMBASE, CNKI). The endpoint was the effects of MSCs on cognitive performance evaluated by the Morris water maze (MWM) test including escape latency, and/or number of platform crossing, and/or time in the target quadrant.

**Results:**

Nine preclinical studies incorporating 225 animals with AD were included for the meta‐analysis. The studies indicated that MSC‐based treatment significantly improved the learning function through measurements of the escape latency (SMD = −0.99, 95% CI = −1.33 to −0.64, *p* < .00001). Additionally, we observed that transplantation of MSCs significantly increased the number of platform crossing in six experiments (SMD = 0.78, 95% CI = 0.43 to 1.13, *p* < .0001). What’s more, the times in the target quadrant were increased in five studies indicated that transplantation of MSCs could ameliorate the cognitive impairments (SMD = 1.06, 95% CI = 0.46 to 1.67, *p* = .0005).

**Conclusions:**

This study showed that MSC transplantation could reduce cognitive deficits in AD models. These findings support the further studies to translate MSCs in the treatment of AD in humans

## 1 INTRODUCTION

Alzheimer’s disease (AD), the leading cause of dementia, is an age‐related neurodegenerative disease. Clinical symptoms include the progressive cognitive function decline, memory loss, and behavior deficit. AD is considered as a major public health concern and a leading cause of disability (Castellani, Rolston, & Smith, 2010; Gjoneska et al., 2015; Stygelbout et al., 2014). According to the data from the European Prevention of Alzheimer’s Dementia (EPAD), currently more than 40 million people worldwide have suffered from AD and its prevalence is expected to double over the next 20 years (Ritchie et al., 2016). Besides, the increasing prevalence of AD represents a global challenge at personal, social, and economic levels (Karran & Hardy, 2014). In 2015, World Alzheimer Report estimated that the total worldwide cost to treat dementia is about $818 billion, and the number is expected to rise to trillion by 2018 (Prince, Wimo, Guerchet, Ali, & Wu, 2015). Unfortunately, today there is no effective therapy to treat or even slow down the progression of AD (Cummings, Morstorf, & Zhong, 2014; Holtzman, Morris, & Goate, 2011; Pharmacology, 2010).Therefore, development of novel treatment strategies for AD is of great clinical significance.

In recent years, different cell replacement therapies have been tested in both animal models and clinical trials and become a promising approach to treat comprehensive human diseases, such as ischemic heart disease, autoimmune diseases, and neurological disorders (Bel et al., 2003; Gratwohl et al., 2010; Kaigler et al., 2013). A number of reports on stem cell transplantation in AD animal models indicate improvement in cognitive and memory performances and increased neuronal survival. Among various stem cells, mesenchymal stem cells (MSCs), a group of multipotent stem cells and immune‐suppressive cells, are the most widely used and offer great promise to treat AD (Caplan & Correa, 2011; Uccelli, Moretta, & Pistoia, 2008). Therapeutic effects of transplantation of MSCs into a murine model of AD have been reported. These studies suggest that the transplantation of MSCs can stimulate neurogenesis in the brains of adult rodents and possibly hinder AD development (Lee, Jin, & Bae, 2010; Massoud & Gauthier, 2010; Shindo, 2006).

However, the source of cells, the administration dose, the site of transplantation, and quality score in each study are so divergent that the overall therapeutic effect is difficult to evaluate. Therefore, the optimal patterns of cell therapy and the actual effects of MSCs on AD remain unclear. In order to clarify the current situation and further studies in MSC therapy as a treatment for AD, we performed this systematic review and meta‐analysis of all available experimental evidence to identify the efficacy of MSC‐based therapies on cognitive impairment in animal models of AD. We will assess the effect of MSC transplantation on cognitive performance by evaluating the performance of various mouse strains in the Morris water maze (MWM) test. The MWM test is a well‐established tool for measuring spatial learning and memory in mouse, and widely used in AD research (Vorhees & Williams, 2006).

## 2 MATERIALS AND METHODS

### 2.1 Systematic search

We performed a systematic review to examine the effects of unmodified MSCs on behavioral outcomes in preclinical AD animal models. The following databases were searched in January 2017: PubMed, MEDLINE, EMBASE, and CNKI, using the terms “Alzheimer’s disease” OR “Alzheimer disease” OR “dementia” AND “mesenchymal stem cell(s) OR “mesenchymal stem cell” OR “MSC” OR “MSCs” AND “animal” OR “animal models.” All the searches were limited to literatures published between January 1980 and January 2017.

### 2.2 Inclusion and exclusion criteria

Studies were included if they met all of the following criteria: (1) use of unmodified MSCs in at least one experimental group; (2) animal models of AD were assigned to either a group for the topical or systemic transplantation of MSCs or a control group (placebo (saline, phosphate‐buffered saline (PBS) or vehicle); (3) use of MWM test to measure behavioral response to treatment; (4) available in English or Chinese language; and (5) original data (not a review).

**Figure 1:**
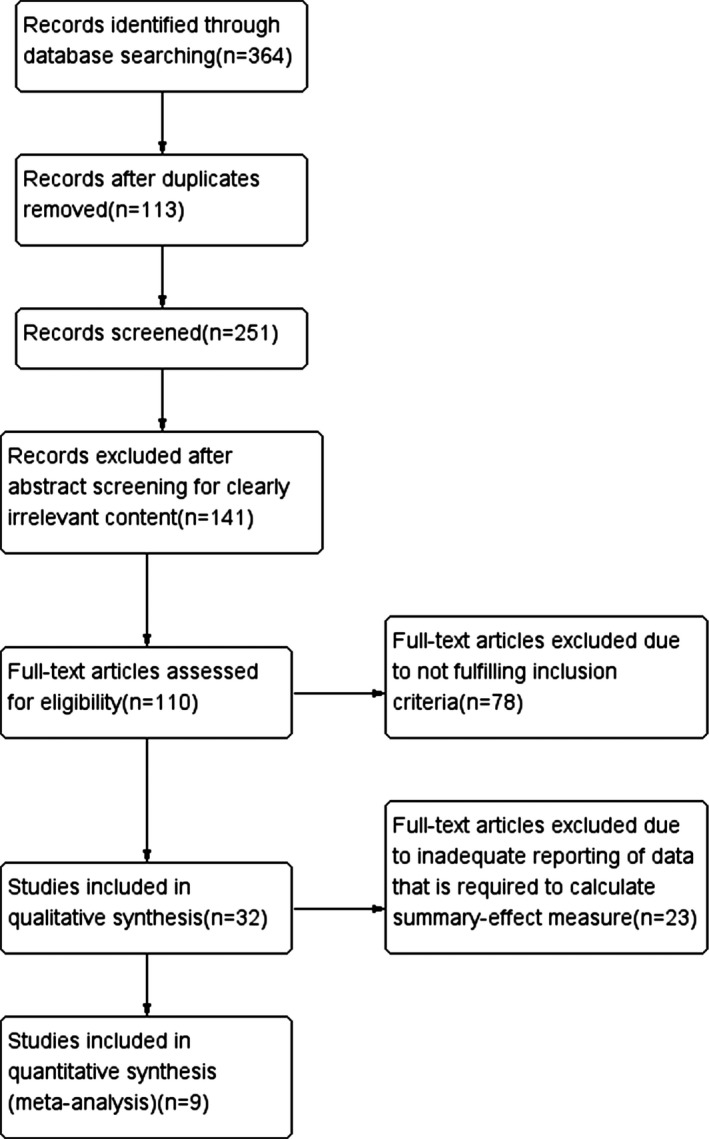
Flow diagram of the search process

We excluded studies testing stem cells other than MSCs and studies with incomplete reporting of data or sample size. The flow of information from identification to inclusion of studies is summarized in Figure 1.

### 2.3 Data extraction

Two authors (Meiling Ge and Yunxia Zhang) independently appraised all titles and abstracts and then full‐text articles. The following information was extracted from text and graphs in each included study: article information(author and publication year), animal species, animal sex, type of AD models, stem cell treatment modalities (source of MSCs, quantity, delivery method used for transplantation, and the duration of the outcome assessment.

The MWM test was used to assess cognition function in all the studies included in this analysis. Detailed methodology is as previously described by Vorhees CV (Vorhees & Williams, 2006). The digital pickup camera was used to record animal behaviors including escape latency, and/or number of platform crossing, and/or time in the target quadrant. When neurobehavioral tests were performed serially, only the final time‐point data were extracted. If data were expressed only graphically, original data were requested from the authors; if a response was not received, data were measured using digital ruler software (Engauge Digitizer 4.1). If one study examined different AD models or MSC doses, then these data were extracted and treated as independent experiments.

### 2.4 Quality assessment

We assessed the quality of the included studies based on a checklist of the Collaborative Approach to Meta‐Analysis and Review of Animal Data from Experimental Studies (CAMARADES), universally applicable to preclinical studies (Zhang, Xing, Ye, Ai, & Wei, 2006). One point was given for evidence of each quality criterion. The quality of all studies was assessed independently by two reviewers.

The criteria included the followings: (1) publication in a peer‐reviewed journal; (2) presence of randomization; (3) the clear characteristics of the (species, background, sex, and age); (4) blinded assessment of behavioral outcome; (5) the specific age at which MSCs were transplanted; (6) the administration route was specified; (7) indication of the number of MSCs; (8) conduction of pretreatment behavioral assessment; (9) statement of potential conflict of interests; and (10) use of suitable animal models (Table 2).

### 2.5 Statistical analysis

According to the Cochrane Handbook for Systematic Reviews of Interventions, we considered the outcomes as continuous data. Continuous outcomes measured on the same scale were expressed as a mean value and SD and were analyzed using standard mean differences (SMD). For cognitive functions (escape latency, number of platform crossing, and time spent in the target quadrant in the MWM test), we quantified the effect of treatment by calculating outcome ratios of the experimental groups to their corresponding control groups. To take the heterogeneity between multistudies into account, a random‐effects model was performed to estimate the combined effect sizes. Q statistic and I‐square (*I* ^*2*^) test were performed to assess the impact of study heterogeneity on the results of the meta‐analysis. *p* value <.1 and *I* ^*2*^ value of >50% were considered statistically significant. Forest plot was generated to depict the SMD along with its 95% confidence interval for each study as well as the pooled mean difference by combining all studies.

Finally, publication bias was explored by funnel plot. All analyses were performed with Stata software (version 5.3, Review Manager).

## 3 RESULTS

### 3.1 Study selection

Among the 363 publications reviewed, 251 potentially relevant papers were screened. Of these, 32 met our inclusion criteria and then 23 studies were excluded due to inadequate reporting of data that is required to calculate summary‐effect measure outcome. Therefore, nine studies investigating the therapeutic effect of MSCs on cognitive deficits in AD animal models were included in the meta‐analysis (Banik, Prabhakar, Kalra, & Anand, 2015; Cui et al., 2017; Fengxian, Wm, & Ling, 2013; Kim et al., 2013; Lee et al., 2012; Lee, Lee, et al., 2010; Li, Rilong, & Yue, 2012; Yang, Yang, et al., 2013; Yang, Yue, et al., 2013). The published studies range from 2010 to 2017.

### 3.2 Study characteristics

Of the nine included studies, two were published in Chinese academic journals and the remainders were published in English. These studies were all preclinical studies in small animal models of AD (mouse). Six studies were performed with transgenic model, and three studies used Aβ‐infused model. Three studies used only males, and the other studies used both genders. Human umbilical cord (four studies) was the most frequently used MSC tissue source, followed by human umbilical cord blood (three studies), and bone marrow and human placenta amniotic membrane (one study, respectively). MSC transplantation was achieved mainly by stereotaxic injection (four studies). Four studies performed tail vein injection, and one study performed intracardiac injection (Table 1).

**Table 1:** Characteristics of included studies

References	Species	Sex	AD model	Type of MSC	Number of MSC injected	Route of administration	Follow‐up period	Outcome Index
Lee, Lee, et al. (2010)	Mouse	M/F	Aβ infused Model	hUCB‐MSC	1 × 10^4^	Stereotaxic(hippocampus)	5 days	1. Escape latency
Lee et al. (2012)	Mouse	M/F	Transgenic Model	hUCB‐MSC	1 × 10^5^	Stereotaxic (hippocampus)	10 days	1. Escape latency2. Number of platform crossing
Li et al. (2012)	Mouse	M/F	Aβ infused Model	BM‐MSC	1 × 10^6^	Injected into the tail vein	5 days	1. Escape latency
Yang, Yue, et al. (2013)	Mouse	M	Transgenic Model	hUC‐MSC	1−2 × 10^6^	Injected into the tail vein	5 days	1. Escape latency2. Number of platform crossing3. Time in the target quadrant
Yang, Yang, et al. (2013)	Mouse	M	Transgenic Model	hUC‐MSC	0.7 × 10^6^	Intracardiac injection	5 days	1. Escape latency2. Number of platform crossing3. Time in the target quadrant
Kim et al. (2013)	Mouse	M/F	Transgenic Model	A‐MSC	2 × 10^6^	Injected into the tail vein	5 days	1. Escape latency
Fengxian et al. (2013)	Mouse	M/F	Transgenic Model	hUC‐MSC	1.5 × 10^6^	Stereotaxic (hippocampus)	4 weeks	1. Escape latency2. Number of platform crossing3. Time in the target quadrant
Banik et al. (2015)	Mouse	M/F	Aβ infused Model	hUCB‐MSC	1 × 10^5^	Stereotaxic (hippocampus)	6 days	1. Escape latency2. Number of platform crossing3. Time spent in the target quadrant
Cui et al. (2017)	Mouse	M	Transgenic Model	hUC‐MSC	2 × 10^6^	injected into the tail vein	7 days	1. Escape latency2. Number of platform crossing3. Time in the target quadrant

M, Male; F, Female; hUCB‐MSCs, human umbilical cord blood‐derived mesenchymal stem cells; hUC‐MSCs, human umbilical cord‐derived mesenchymal stem cells; BM‐MSC, bone marrow‐derived mesenchymal stem cells; A‐MSC, amniotic mesenchymal stem cells.

Although all the included studies used MWM test to assess cognitive function, among these studies different subtests were used: All the studies measured escape latency; six studies measured the number of platform crossing to assess spatial learning function; five studies assessed the spatial learning function through measuring the time in the target quadrant.

### 3.3 Methodological quality of studies

Table 2 shows the methodological quality of the enrolled studies. The score of the included studies quality ranged from 8 to 12 of a total 13 points. Four studies reported the randomization of animals into treatment groups, but did not mention the method of randomization. All studies stated the potential conflict of interests. All the studies showed that outcome measurements were assessed by computer program which was blind to the treatment conditions. Moreover, no studies described the sample size calculation to confirm that sufficient power had been achieved. The lowest score was eight items (11.11%), and the highest score was 12 items (11.11%).

**Table 2:** Quality assessment of the included studies

References	1	2	3				4	5	6	7	8	9	10	Quality score (items)	Quality score (%)
			a	b	c	d									
Hyun (2010)	√	No	√	√	No	√	√	√	√	√	√	√	√	11	44.4
Hyun (2012)	√	No	√	√	No	√	√	√	√	√	No	√	√	10	33.3
Li et al. (2012)	√	√	√	√	√	√	√	No	√	√	√	√	√	12	11.1
Yang, Yue, et al. (2013)	√	No	√	√	No	No	√	No	√	√	No	√	√	8	11.1
Yang, Yang, et al. (2013)	√	No	√	√	√	√	√	No	√	No	√	√	√	10	33.3
Kyung (2013)	√	No	√	√	√	√	√	√	√	√	No	√	√	11	44.4
Sun (2013)	√	√	√	√	No	√	√	√	√	√	No	√	√	11	44.4
Avijit (2015)	√	No	√	√	√	√	√	No	√	√	No	√	√	10	33.3
Cui et al. (2017)	**√**	**√**	**√**	**√**	**No**	**No**	**√**	**√**	**√**	**√**	**√**	**√**	**√**	**11**	**44.4**

√ = fulfilling the criterion, no = not fulfilling the criterion. 1: publication in a peer‐reviewed journal; 2: presence of randomization; 3: the clear characteristics (a:species; b:background;c:sex;d:age) of the study population; 4: blinded assessment of behavioral outcome; 5: the specific age at which mesenchymal stem cells (MSCs) were transplanted; 6: the route of administration was specified; 7: the number of MSCs were mentioned; 8: pretreatment behavioral assessment was conducted; 9: statement of potential conflicts of interest; and 10: use of suitable animal models.

### 3.4 Meta‐analysis

**Figure 2:**
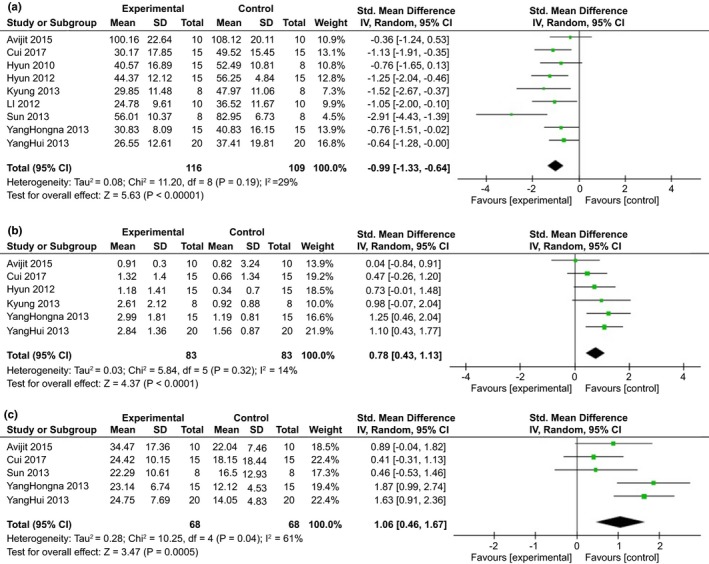
Forest plot showing the impact of mesenchymal stem cell (MSC) on cognitive deficits, compared with controls, according to (a) escape latency time, (b) number of platform crossings, (c) time in target quadrant

All the data for meta‐analysis were expressed graphically, and the Engauge Digitizer 4.1 was used to calculate the mean and standard error. The effects of MSC transplantation on cognitive deficits in AD models using the MWM test were examined in nine comparisons of nine included studies involving 225 animals. Nine studies reported the effect of MSC transplantation on decreasing escape latency compared with the control group (Banik et al., 2015; Cui et al., 2017; Fengxian et al., 2013; Kim et al., 2013; Lee et al., 2012; Li et al., 2012; Peng, Xing, Yang, Wang, & Wang, 2014; Yang, Yang, et al., 2013; Yang, Yue, et al., 2013).The pooled analysis indicated significant effectiveness in the ability of learning through measurements of the escape latency, which was the time that the mice in the maze successfully found the hidden platform (SMD = −0.99, 95% CI = −1.33 to −0.64, *p* <.00001). There was no heterogeneity among studies (*I* ^2^ = 29%, *p* = .19) (Figure 2a).

Additionally, six studies reported the impact of MSC transplantation on increasing number of platform crossing compared with the control group (Banik et al., 2015; Cui et al., 2017; Fengxian et al., 2013; Kim et al., 2013; Li et al., 2012; Peng et al., 2014; Yang, Yang, et al., 2013; Yang, Yue, et al., 2013). We observed that transplantation of MSCs significantly increased the number of platform crossing in six experiments (SMD = 0.78, 95% CI = 0.43 to 1.13, *p* < .0001).The outcome that had no heterogeneity (*I* ^2^ = 14%, *p* = .32) was observed across the studies (Figure 2b).

What’s more, five studies reported the impact of MSC transplantation on increasing time in target quadrant compared with the control group. (Banik et al., 2015; Kim et al., 2013; Peng et al., 2014; Yang, Yang, et al., 2013), and the data indicated significant effectiveness of MSCs compared with the control group (SMD = 1.06, 95% CI = 0.46 to 1.67, *p* = .0005. heterogeneity, *I* ^2^ = 61%, *p *= .04) (Figure 2c).

### 3.5 Stratified analysis

To further explore the potential influence of study design on the beneficial effect of MSCs on AD, we further performed stratified analyses based on animal gender, type of AD model, type of MSCs, route of admission, and doses of MSCs. The results of the stratified analyses are described in Table 3.

**Table 3:** The results of stratified meta‐analysis

Subgroups	Escape latency time			*p* **	Number of platform crossing			*p* **	Time in the target quadrant			*p* **
	studies	SMD [95% CI]	*p* *		studies	SMD [95% CI]	*p* *		Studies	SMD [95% CI]	*p* *	
Gender												
Male	3	−0.81 [−1.22, −0.40]	.0001	.33	3	0.93 [0.47, 1.39]	<.0001	.30	3	1.29 [0.39, 2.19]	.005	.30
Mix	6	−1.15 [−1.71, −0.60]	.09		3	0.56 [0.04, 1.09]	.04		2	0.69 [0.01, 1.37]	.05	
Type of AD model												
Aβ infused Model	3	−0.71 [−1.23, −0.18]	.008	.22	1	0.04 [−0.84, 0.91]	.93	.07	1	0.89 [−0.04, 1.82]	.06	.73
Transgenic Model	6	−1.15 [−1.62, −0.68]	<.00001		5	0.90 [0.55, 1.24]	<.00001		4	1.10 [0.35, 1.85]	.004	
Type of MSC												
hUC‐MSC	4	−1.12 [−1.79, −0.44]	.001	.61	3	0.93 [0.47, 1.39]	<.0001	.45	4	1.10 [0.35, 1.85]	.004	NA
hUCB‐MSC	3	−0.82 [−1.34, −0.30]	.002		2	0.43 [−0.25, 1.10]	.22		1	1.06 [0.46, 1.67]	.06	
BM‐MSC or A‐MSC	2	−1.24 [−1.97, −0.51]	.0009		1	0.98 [−0.07, 2.04]	.07		0	NA	NA	
Route of administration												
Stereotaxic	4	−1.16 [−1.99, −0.33]	.006	.78	2	0.43 [−0.25, 1.10]	.22	.30	2	0.69 [0.01, 1.37]	.05	.11
Injected into the tail vein	4	−0.96 [−1.37, −0.55]	<.00001		3	0.84 [0.39, 1.29]	.0002		2	1.02 [−0.18, 2.22]	.09	
Intracardiac injection	1	−0.76 [−1.51, −0.02]	.05		1	1.25 [0.46, 2.04]	.002		1	1.87 [0.99, 2.74]	<.0001	
Doses of MSC												
≥1 × 106	5	−1.24 [−1.83, −0.64]	<.0001	.24	3	0.84 [0.39, 1.29]	.0002	.72	3	0.86 [0.03, 1.70]	.04	.41
<1 × 106	4	−0.81 [−1.22, −0.40]	.0001		3	0.70 [0.04, 1.35]	.04		2	1.39 [0.44, 2.35]	.004	

p*, value for heterogeneity within each subgroup; p**, value for heterogeneity between subgroups with meta‐regression analysis; hUC‐MSC, human umbilical cord‐derived mesenchymal stem cells; hUCB‐MSC, human umbilical cord blood‐derived mesenchymal stem cells; BM‐MSC, bone marrow‐derived mesenchymal stem cells; A‐MSC, amniotic mesenchymal stem cells.

Firstly, the protective effects of MSCs on escape latency were examined. After treatment, escape latency was remarkable improved in both stereotaxic injection of MSCs (SMD = −1.16, 95% CI: −1.99 to −0.33, *p *= .006) and intravenous injection (SMD = −0.96, 95% CI: −1.37 to −0.55, *p* < .00001). However, intracardiac injection of MSCs did not significantly affect the escape latency in animals of AD (SMD = −0.76, 95% CI: −1.51–0.02; *p* = .05). Other study characteristics, such as the type of AD models, the type of MSCs, and doses of MSCs appeared to have no significant influence on the benefits of MSC transplantation on escape latency. Secondly, we examined the effects of MSCs on examined the number of platform crossing. Doses of MSCs make no difference on increasing the number of platform crossing. Among the transgenic AD model, significant beneficial effects were found (SMD = 0.90, 95% CI: 0.55 to 1.24, *p* < .00001) (Table 3).

### 3.6 Publication bias

**Figure 3:**
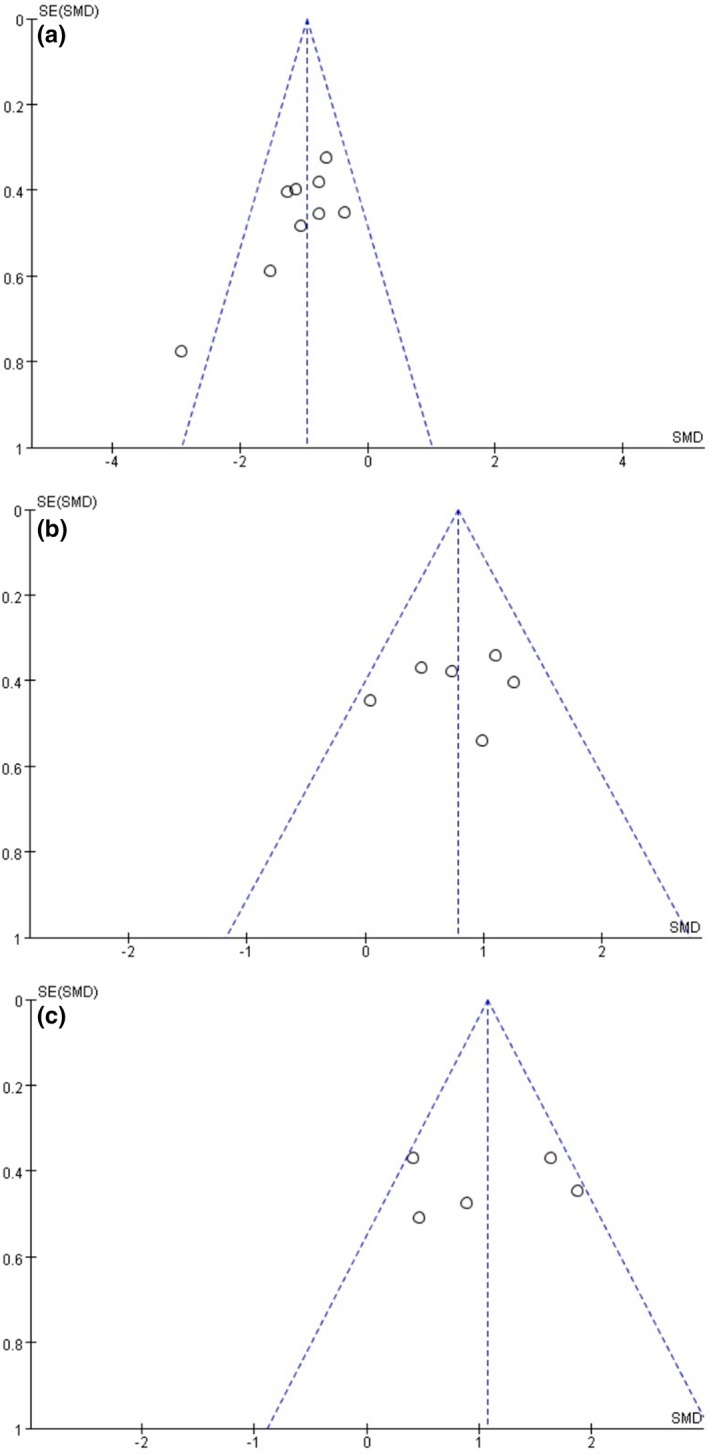
Funnel graph for the assessment of potential publication bias of the effect of MSC on cognitive deficits, according to (a) escape latency time, (b) number of platform crossings, (c) time in the target quadrant

No evident publication bias for the effect of acupuncture on escape latency, the number of platform crossing, and time spent in the target quadrant using the MWM test (Figure 3a,b,c) was obtained through the visual distribution of funnel plot. Nevertheless, the use of funnel plot was limited for the outcomes of MWM test due to the small number of studies evaluated.

## 4 DISCUSSION

Systematic reviews and meta‐analyses of animal studies provide a more objective evidence for researchers assessing the advantages and effects of experimental interventions before they decide to proceed with clinical trials or not. This is the first systematic review and meta‐analysis to examine the efficacy of MSC transplantation for improving cognitive function in animal models of AD with the results of MWM test as the outcome measures (Liao, Zhang, Li, Shen, & Zhong, 2014; Peng et al., 2014). Overall, our study suggested that MSCs has the neuroprotective effects in improving cognitive outcomes in AD and the effects were robust across species, delivery route, type of MSCs, and MSC dose. The results showed that MSC transplantation could significantly reduce the time of escape latency, decrease the number of cross platform, and increase the proportion of time spent in the target quadrant in animal models of AD with cognitive deficits. We regarded functional recovery and behavioral testing as outcomes of this analysis because it is a common parameter widely used to measure functional disability and recovery in animal models of neurological disorders (Kirik, Rosenblad, & Rklund, 1998; Olsson, Nikkhah, Bentlage, & Bjorklund, 1995). These findings support further translational studies of MSCs in the treatment of AD in humans.

Although our study demonstrates that MSC transplantation could improve cognitive function, the underlying mechanism remains unclear. Several studies have assessed MSCs as therapeutic agents to reverse pathological changes or induce neurogenesis in animal models of AD (Chen et al., 2014; Garcia et al., 2014). Firstly, previous studies verified that the neuroprotective and neurogenesis effect of MSCs was related to the release of neurotrophins such as acetylcholine (Ach) and nerve growth factor (NGF) (Fahnestock, Garzon, Holsinger, & Michalski, 2002; Fumagalli, Racagni, & Riva, 2006; Peng, Wuu, Mufson, & Fahnestock, 2005; Siegel & Chauhan, 2000) .In addition, MSCs also upregulate the expression of the anti‐apoptotic factors to protect neurons. Secondly, many evidences proved that MSCs attenuate the syndrome of AD and prevent the progression of the disease by expressing antioxidant enzymes, alleviating oxidative stress (Chiang, Nicol, Cheng, Lin, & Yen, 2016; Ruzicka, Kulijewicz‐Nawrot, Rodrigez‐Arellano, Jendelova, & Sykova, 2016; Xie et al., 2016; Yang, Xie, et al., 2013). Thirdly, inhibition of activated microglia and decreased levels of Aβ plays a critical role in MSC‐induced cognitive improvement (Woodruffpak, 2008). What’s more, MSCs exerted significantly immune‐suppressive function and anti‐inflammatory effect may be associated with improved cognitive deficit of AD animal model. The underlying mechanism of MSC transplantation ameliorating cognition deficits is complex. In a word, more underlying mechanisms of this phenomenon should be investigated in the future.

There are some limitations in our study. Firstly, animal model of AD may not fully recapitulate all aspects of cognitive function development observed in humans with MSCs, as a result, it will limit the extent to which this experimental research translates to a clinical population. Secondly, potential publication bias is likely to exist although we had made an extensive effort to identify all the relevant studies, our analysis was only able to include data from the published studies in this field. Our analysis did not take unpublished data into account, so our study might overestimate the overall effect size (Schmucker et al., 2013). Another limitation is that the data were reported in the form of graph, and we extracted the data using the Engauge Digitizer 4.1. Furthermore, behavior tests in animal models of AD cannot fully represent all the components of neurological condition of AD. Finally, all the studies which were included in our meta‐analysis used small animal models (mouse) of AD. Therefore, randomized and blinded controlled studies in large animal models of AD are warranted.

## 5 CONCLUSIONS

Our present systematic review with meta‐analysis indicates that transplantation of MSCs improves cognitive function in animal models of AD. These results suggest that MSC‐based strategies may become an alternative treatment for AD. Although trials for MSC therapy have been performed primarily in small animals, in order to assess the efficacy and safety of MSCs on cognitive deficits, more studies in preclinical animal models and human studies, randomized controlled design, are needed.

## CONFLICT OF INTEREST

The authors declared that they have no conflict of interest.
